# Long-term outcomes after revascularization surgery for adult moyamoya disease: Protocol for systematic review and meta-analysis

**DOI:** 10.1371/journal.pone.0318370

**Published:** 2025-04-17

**Authors:** Sha Wang, Jingjin Zhao, Xinzhu Chen, Liangzhen Zhou, Wenyao Cui

**Affiliations:** Department of Neurosurgery, West China Hospital, Sichuan University, Chengdu, People’s Republic of China; Coventry University, UNITED KINGDOM OF GREAT BRITAIN AND NORTHERN IRELAND

## Abstract

**Introduction:**

Moyamoya disease (MMD) commonly presents with cerebral ischemia or hemorrhage. Revascularization surgery, including direct, indirect, and combined bypasses, remains the mainstay of MMD treatment aiming to reduce stroke recurrence. However, the long-term outcomes of these interventions are still controversial. Herein, we designed the protocol for a systematic review and meta-analysis comparing the three different revascularization surgeries in adult MMD patients for more than 5 years long-term outcomes.

**Methods:**

Our systematic review and meta-analysis protocol follows the PRISMA-P guidelines. Bypass surgeries for MMD will be included only if the follow-up time is more than 5 years. The primary outcome is the incidence of recurrent cerebral ischemia or hemorrhage. The secondary outcomes include functional outcomes evaluated by the Moyamoya Disease outcome category, mortality, and angiographic outcomes such as bypass patency, and collateral formation.

**Results:**

Studies on the postoperative outcomes of revascularization surgeries to treat adult MMD patients will be included and analyzed. This systematic review and meta-analysis will offer evidence to clinicians and researchers on the long-term outcomes of revascularization surgeries in adult MMD patients and help to optimize the selection of bypass modality.

## Introduction

Moyamoya disease (MMD), also known as spontaneous steno-occlusion of the circle of Willis, is a cerebrovascular disease characterized by slow progressive narrowing leading to occlusion of the bilateral terminus of the internal carotid arteries and/or the initial segments of the anterior and middle cerebral arteries, with the appearance of compensatory abnormal vascular networks at the base of the brain [1,2]. The annual incidence of moyamoya disease ranges from 0.5 to 1.5 per 100,000 individuals in East Asian nations, while it is as low as 0.1 per 100,000 in other regions [3]. Due to the unclear pathogenesis of MMD, there is a current absence of specific pharmacological treatments available for MMD. Antiplatelet therapy is one of the most promising treatment options but randomized controlled trials are still necessary to confirm its effectiveness [4]. Thus, surgical procedures for intracranial and extracranial blood flow reconstruction remain the mainstay of clinical intervention for MMD [5].

Despite several studies addressing the efficacy of bypass surgery in mitigating recurrent strokes among adult moyamoya patients, the benefits of these interventions remain controversial [6–8]. The notion that revascularization procedures effectively enhance cerebral blood flow, thereby reducing the incidence of ischemic strokes, has been widely accepted by most scholars. However, a recent review denoted that surgery is not superior to medical management in patients presenting with cerebral ischemia [9]. JAM trial is the only single randomized control study of MMD, that compared revascularization with conservative treatment in patients with hemorrhagic MMD, showing substantially fewer recurrent hemorrhages in surgical patients [10]. The trial was criticized for unmasked outcome evaluation, low enrollment, and no postoperative complication which was too good to be true [11]. Several meta-analyses have been performed to elucidate the beneficial effects of revascularization surgery [12–16]. The limitations of these meta-analyses were pointed out by Moussouttas et al. [9], such as no specific data regarding the outcome types of hemorrhagic or ischemic events, and no mortality information. Moreover, the follow-up period varied greatly for each included study of meta-analysis, making outcome assessments difficult to compare.

It was concluded that the natural history of hemorrhagic MMD remains dynamic with cumulative risk of rebleeding being 7.8% at 5 years, 22.6% at 10 years, and 35.9% at 15 years, requiring long-term follow-up to fully understand the risk of rebleeding [17]. Similarly, Morioka et al. [18] reported that there is a higher risk of rebleeding in the first 6 years after initial hemorrhage. In 2016, a meta-analysis attempted to determine the best surgical management for adults with MMD by comparing long-term outcomes but with just more than 30 days of follow-up after surgery among direct, indirect, and combined bypass types [19]. In 2018, a long-term good functional outcome rate of 82% was achieved after revascularization in a meta-analysis where the long-term follow-up time was defined as at least 6 months [16]. Most of the existing systematic reviews defined events after 30 days or 6 months as late outcomes, but long-term retrospective studies have demonstrated that the incidence of adverse events is gradually increasing until 36 months after surgery, and some studies have reported that different surgical methods appear late crossovers on the bleeding free survival curve at 36 months after surgery. The considerable heterogeneity and inconsistent definitions of long-term outcomes may result in a lack of overall credibility in these findings. In a 10-year follow-up study, investigators observed a higher frequency of rebleeding cases during the 5–10 years after indirect revascularization for adult hemorrhagic moyamoya disease compared with the first 5 years after the procedure [20]. The long-term follow-up results after combined revascularization surgery also indicated a decreasing trend in cerebral blood flow in the territory of the middle cerebral artery (MCA) in the operated hemispheres as the follow-up period progresses [21]. Overall, the current results strongly suggest that the evaluation of postoperative outcomes following revascularization surgery should be extended to 5 years or even longer to reflect the long-term benefits or risks for patients. However, there is still insufficient evidence regarding long-term follow-up.

We design to conduct a systematic review and meta-analysis to clarify the long-term angiographic and functional outcomes with at least 5 years of follow-up under the uniform outcome measurement tools. Direct, indirect, and combined bypasses will be compared regarding the specific hemorrhagic or ischemic events or mortality rate in adults with MMD. Accordingly, we aim to summarize existing knowledge regarding the effect of surgery on MMD outcomes and guide the selection of surgical approaches to achieve long-term comprehensive clinical outcomes for patients.

This systematic review aims to assess the postoperative long-term outcomes after revascularization surgery of MMD in adult patients (aged 18 or older, with no limitations on sex and race). We seek to answer the following questions:

What is the overall incidence of angiographic and functional outcomes among adult patients who underwent revascularization surgery of MMD in the postoperative long-term period?

Is there a trend in the incidence of adverse events after surgery over time or in different regions of the world?

What is the relevance between revascularization bypass types and postoperative long-term adverse events among adult patients with MMD, and how to determine the best surgical management for adults with MMD?

## Materials and methods

**Fig 1:**
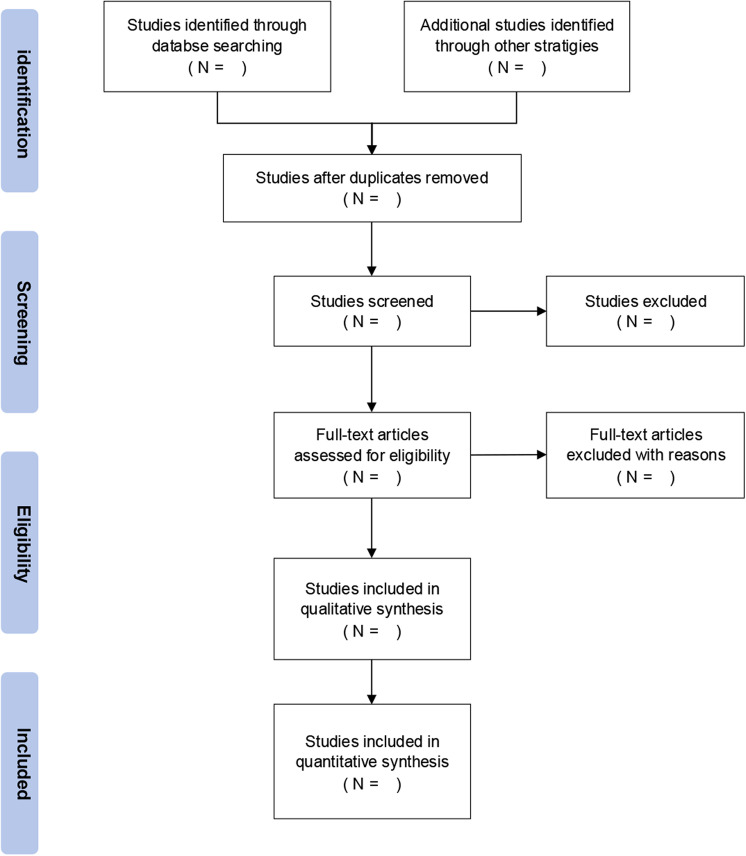
PRISMA flow diagram.

Our systematic review and meta-analysis protocol follows the PRISMA-P guidelines (S1 Table) [22]. This protocol has been registered on the PROSPERO (registration number ID: CRD42024550222). Since this investigation will not involve disclosure of patient information, ethics approval is waived. The PRISMA flow diagram will be used to record every step of the review process (Fig 1).

### Eligibility criteria

#### Types of study.

Randomized controlled trials (RCT), observational studies (cohort studies, and case-control studies [CCS]) will be included while case reports and letters to editors will be excluded.

#### Types of participants.

We will include adult patients diagnosed with MMD according to the current guideline, including those without a history of stroke or transient ischemic attack (TIA). We will exclude patients with moyamoya syndrome, i.e., ICA stenosis or occlusion due to known reasons, for example, atherosclerosis, trauma, or radiation. Due to possible variations in disease diagnosis, the definition of “MMD” will be extracted from included studies.

#### Types of interventions.

Patients underwent revascularization surgery either by direct bypass, indirect bypass, or combined bypass.

#### Types of outcome measures.

Primary outcomes: Recurrent ischemia, Intracranial Hemorrhage
Secondary outcomes: Functional outcome (measured by the Moyamoya Disease outcome category proposed by Kim et al.), Mortality, Angiographic bypass patency, Angiographic collateral formation

### Search strategies

We will search the following five electronic databases: PubMed, Cochrane Library, MEDLINE Ovid, Embase Ovid, and Web of Science. The literature search will include studies published from the inception of the database to the date of the commencement of the review. Table 1 shows the initial draft search strategy for PubMed. The search strategies for other databases are shown in S1 Appendix. In addition, we will use the Cited Reference Search within Web of Science to check the bibliographies of included studies and any relevant systemic reviews for further references to relevant trials and search Google Scholar (scholar.google.co.uk/) to forward-track relevant references. If trial reports are unclear, we will contact the original authors for clarification and further data.

**Table 1:** Search strategy for PubMed.

Search	Query
#1	((moyamoya disease) OR (moyamoya[Text Word])) OR (moya-moya[Text Word])
#2	(adult) OR (adults)
#3	“outcome”[Title/Abstract] OR “outcomes”[Title/Abstract] OR “effectiveness”[Title/Abstract] OR “efficacy”[Title/Abstract] OR “rehabilitation”[Title/Abstract] OR “prognosis*”[Title/Abstract] OR “factor*”[Title/Abstract]
#4	“surgical” OR “surgery” OR “cerebral revascularization” OR “indirect bypass” OR “indirect revascularization” OR “direct bypass” OR “direct revascularization” OR “revascularization” OR “superficial temporal artery middle cerebral artery anastomosis” OR “encephalomyosynangiosis” OR “encephaloduroarteriosynangiosis” OR “encephaloduroarteriogaleosynangiosis” OR “encephaloduroarteriomyosynangiosis” OR “combined bypass” OR “direct anastomosis” OR “indirect anastomosis” OR “superficial temporal artery to middle cerebral artery” OR “STA-MCA” OR “STA-MCA bypass” OR “encephalo-duro-arterio-synangiosis” OR “EDAS” OR “encephalo-myo-synangiosis” OR “EMS” OR “encephalo-duro-arterio-myo-synangiosis” OR “multiple burr holes” OR “pial synangiosis”
#5	#1 AND #2 AND #3 AND #4
Filter: before the date of the commencement of the review	

#### Inclusion criteria.

Patients with MMD older than 18 years (the diagnostic criteria of MMD must be consistent with that of the original literature RCPTSOCW 2012 [5]);
Patients who underwent revascularization surgery either by direct bypass, indirect bypass, or combined bypass;
Articles reporting prespecified perioperative long-term outcomes after surgery at least more than 5 years;
Data should involve estimates of odds ratio (OR), relative risk (RR), or hazard ratio (HR) with 95%confidence interval (CI);
Reported in English or Chinese

#### Exclusion criteria.

It was an editorial, a review, a case report, or a cross-sectional study;
Did not separate pediatric from adult patients;
Did not report our outcomes of interest;
Studies lacking specific data for adult group or presenting data is difficult to extract (e.g., presented by line charts);
The enrolled patients had other intracranial diseases;
MMD was managed conservatively.

The results of the literature search will be imported into an EndNote V.X9.3.3 database, and duplicates will be removed. We will next establish several independent groups for each selecting stage in the EndNote database. Two independent investigators will screen the literatures in two stages. At first, they will review the titles and abstracts retrieved from database searches to evaluate all studies that meet the inclusion criteria. Next, they will retrieve the full-text articles for the remaining references and review authors will independently screen the full-text articles, identify studies for inclusion, and identify and record reasons for exclusion of the ineligible studies. All discrepancies will be resolved by consultation with a third investigator. For studies that share the same data source, we will prioritize studies with larger sample size and longer study durations in the meta-analysis.

### Data extraction

We will design a form to extract data which will include the following details: (1) study authors; (2) research period and publication year; (3) study design; (4) country and continent of the study; (5) data source; (6) study population and inclusion criteria; (7) definition of “adult” and diagnostic criteria for “MMD”; (8) number of patients included in the adult group; (9) preoperative Suzuki angiographic stage of hemispheres; (10) outcome measurement; (11) follow-up time; (12) rates of prespecified long-term outcomes. Two review authors will extract data using the agreed form. Discrepancies will be solved through discussion. Data are about to be entered into Review Manager software (RevMan 2014) and checked for accuracy.

The long-term outcome measures include rates of long-term angiographic or functional outcomes events occurring more than 5 years after bypass and rates of favorable outcomes at the last follow-up. Definitions of favorable outcomes varied among studies; both clinical improvement and angiographic improvement will be included in our definition of favorable outcomes.

### Quality assessment

In this study, we will use the guidance from the Grading of Recommendations Assessment, Development, and Evaluation (GRADE) working group to assess the quality of evidence for the primary outcome. The GRADE summary of findings table will be produced using the GradePRO software. We will evaluate evidence quality based on the domains of risk of bias, consistency, directness, precision, and publication bias. Additional domains may be considered as needed. Quality will be categorized as high, moderate, low, or very low.

To assess the risk of bias for RCTs and non-RCTs, the Risk Of Bias In Non-randomised Studies - of Interventions (ROBINS-I) tool and a revised tool for assessing risk of bias in randomised trials (RoB 2) will be used. For non-RCTs, evaluators will assess the level of bias risk in 7 domains in ROBINS-I and give a risk assessment of “low, moderate, serious, critical, or no information” for each domain. Based on the assessment of all individual domains, an overall assessment of “overall bias” will be made according to the following rules: if all seven domains have a low bias risk, the overall bias risk is “low”; if all seven domains have a low or moderate risk of bias, the overall bias risk is “moderate”; if at least one domain has a high risk of bias but no evaluation field has an extremely high risk of bias, the overall bias risk is “high”; if at least one domain has an extremely high risk of bias, the overall bias risk is “critical”; if there is no relevant information on key domains, the overall bias risk is “no information”. With respect to RCTs, each domain in RoB 2.0 will be characterized as at low risk of bias; causing some concerns; or at high risk of bias. If the assessment results in all five domains are low risk, the study will be determined as “low risk of bias”; if any domain report a high risk or multiple domains report “causing some concerns”, it is determined as “high risk of bias”; RCTs other than the above two situations are determined as “some concerns”.

Publication bias will be assessed using contour-enhanced funnel plots. The presence of missing studies in areas of the plot without significant differences suggests that the funnel plot asymmetry may be due to publication bias; the presence of missing studies in areas with significant differences suggests that the asymmetry may be due to other causes, such as heterogeneity, rather than publication bias.

### Strategy for data synthesis

We will carry out statistical analysis using the Review Manager software (RevMan 2014). Heterogeneity will be assessed using the I² statistic, with values ≤ 50% being regarded as indicating low heterogeneity, in which case, we will use fixed-effect meta-analysis for combining data where it is reasonable to assume that studies were estimating the same underlying treatment effect: i.e., where trials were examining the same intervention, and the trials’ populations and methods were judged sufficiently similar. Values of I² > 50%, however, are considered to indicate significant heterogeneity, in which case we will use random-effects meta-analysis to produce an overall summary and conduct subgroup analyses to investigate the sources of the heterogeneity. For studies with highly significant heterogeneity (I² > 75%), we will conduct a manual review to identify potential issues such as small sample size, study design flaws, or poor data quality. If such issues are identified, the study will be excluded, and the overall heterogeneity will be assessed again. Sensitivity analysis will be performed by removing each study individually to assess the consistency and quality of the results regarding different surgical procedures and the incidence of primary outcomes.

### Statistical analysis

The dichotomous variables outcomes will be assessed as an odds ratio (OR) and 95% confidence interval (CI). Kim et al. categorized Moyamoya Disease favorable functional outcomes into Excellent (symptoms resolved without neurological deficits) and Good (symptoms resolved with neurological deficits) [23]. In this study, single group rates of these favorable outcomes will undergo variance-stabilizing Freeman-Tukey arcsine transformation and transformed rates and errors will be re-converted for meta-analysis, generating mean weighted probability (MWP) and 95% CI in a random-effects model [16]. In the subgroup analyses, we will firstly group studies according to different characteristics of the original study (control set, severity of disease, comorbidities, etc.), compare the combined effect size between the groups, and test for inter-group and intra-group heterogeneity. If there are sufficient studies included, we will next undertake subgroup analyses for the primary outcomes by ethnicity, age groups, preoperative Suzuki stages, different subtypes of MMD, surgical procedure. P value < 0.05 will be considered significant for all analyses.

## Discussion

MMD remains a disease of low incidence, especially in Western countries. There are huge heterogeneities regarding the age, population, clinical presentation, and possible prognosis between western and eastern regions [4]. In addition, surgical modality of revascularization varied across different medical centers with inconsistent efficacy reported. Therefore, it is quite urgent but difficult to perform prospective or even randomized control studies to illustrate the best treatment.

The long-term treatment efficacy of bypass surgery depends on the hemodynamic change and bypass geometry. Computational fluid dynamics (CFD) simulation is a simple, low-cost, noninvasive tool in evaluating the efficacy of STA-MCA bypass from hemodynamic perspectives [24]. It was reported that bypass vessel remodeling occurred, resulting in an increased driving pressure and greater blood flow through the bypass after surgery in most cases [25]. Occasionally, bypass geometry variations, such as tortuosity and stenosis in STA, could significantly increase flow resistance, reducing the distal branch flow rate [26]. However, postoperative stenosis of the STA-MCA bypass is not necessarily negative. The postoperative improvement of distal hemodynamic environment can lead to the generation of collaterals, which might take over bypass function in the long term [27]. The efficacy and outcome of revascularization have been investigated in previous meta-analyses. However, to the best of our knowledge, no systematic review was reported to focus on the long-term outcome beyond 5 years follow-up which is crucial in the precise and comprehensive prognostic evaluation as the chronic hemodynamic changes of MMD and the recurrence of hemorrhage or infarction remain versatile after surgery [28]. We believe our data will be of vital importance in future clinical practice and research.

This study has several limitations. First, the pooled analysis will include both prospective and retrospective studies, the resulting bias in the retrospective studies might impact our overall conclusions. In addition, patients’ conditions on admission are not matched among the different groups, which might have biased our results. Besides, indications for revascularization surgeries could not be standardized.

## Supporting information

S1 Appendix Detailed search strategies. (DOCX)

S1 Table PRISMA-P 2015 checklist: recommended items to address in a systematic review protocol. (PDF)

## References

[1] Suzuki J, Takaku A. Cerebrovascular “moyamoya” disease. Disease showing abnormal net-like vessels in base of brain. Arch Neurol. 1969;20(3):288–99. doi: 10.1001/archneur.1969.00480090076012 5775283

[2] Scott R, Smith E. Moyamoya disease and moyamoya syndrome. N Engl J Med. 2009;360(12):1226–37. 19297575 10.1056/NEJMra0804622

[3] Uchino K, Johnston S, Becker K, Tirschwell D. Moyamoya disease in Washington State and California. Neurology. 2005;65(6):956–8. 16186547 10.1212/01.wnl.0000176066.33797.82

[4] Ihara M, Yamamoto Y, Hattori Y, Liu W, Kobayashi H, Ishiyama H, et al. Moyamoya disease: diagnosis and interventions. Lancet Neurol. 2022;21(8):747–58. doi: 10.1016/S1474-4422(22)00165-X 35605621

[5] Research Committee on the Pathology and Treatment of Spontaneous Occlusion of the Circle of Willis, Health Labour Sciences Research Grant for Research on Measures for Infractable Diseases. Guidelines for diagnosis and treatment of moyamoya disease (spontaneous occlusion of the circle of Willis). Neurol Med Chir (Tokyo). 2012;52(5):245–66. doi: 10.2176/nmc.52.245 22870528

[6] El Naamani K, Chen C-J, Jabre R, Saad H, Grossberg J, Dmytriw A, et al. Direct versus indirect revascularization for moyamoya: a large multicenter study. J Neurol Neurosurg Psychiatry. 2023. 10.1136/jnnp-2022-329176 37673641

[7] Kazumata K, Ito M, Tokairin K, Ito Y, Houkin K, Nakayama N, et al. The frequency of postoperative stroke in moyamoya disease following combined revascularization: a single-university series and systematic review. J Neurosurg. 2014;121(2):432–40. doi: 10.3171/2014.1.JNS13946 24605834

[8] Nguyen VN, Motiwala M, Elarjani T, Moore KA, Miller LE, Barats M, et al. Direct, indirect, and combined extracranial-to-intracranial bypass for adult moyamoya disease: an updated systematic review and meta-analysis. Stroke. 2022;53(12):3572–82. doi: 10.1161/STROKEAHA.122.039584 36134563

[9] Moussouttas M, Rybinnik I. A critical appraisal of bypass surgery in moyamoya disease. Ther Adv Neurol Disord. 2020;13:1756286420921092. doi: 10.1177/1756286420921092 32547641 PMC7273549

[10] Miyamoto S, Yoshimoto T, Hashimoto N, Okada Y, Tsuji I, Tominaga T, et al. Effects of extracranial-intracranial bypass for patients with hemorrhagic moyamoya disease: results of the Japan adult moyamoya trial. Stroke. 2014;45(5):1415–21. doi: 10.1161/STROKEAHA.113.004386 24668203

[11] Esposito G, Amin-Hanjani S, Regli L. Role of and indications for bypass surgery after carotid occlusion surgery study (COSS)? Stroke. 2016;47(1):282–90. doi: 10.1161/STROKEAHA.115.008220 26658449

[12] Jeon JP, Kim JE, Cho W-S, Bang JS, Son Y-J, Oh CW. Meta-analysis of the surgical outcomes of symptomatic moyamoya disease in adults. J Neurosurg. 2018;128(3):793–9. doi: 10.3171/2016.11.JNS161688 28474994

[13] Yan Y, Li Y, Huang L, Zhang S. a comprehensive meta-analysis for bypass surgery in adult moyamoya. World Neurosurg. 2019;124:161–70. doi: 10.1016/j.wneu.2018.12.183 30654155

[14] Wouters A, Smets I, Van den Noortgate W, Steinberg GK, Lemmens R. Cerebrovascular events after surgery versus conservative therapy for moyamoya disease: a meta-analysis. Acta Neurol Belg. 2019;119(3):305–13. doi: 10.1007/s13760-019-01165-9 31215004

[15] Li Q, Gao Y, Xin W, Zhou Z, Rong H, Qin Y, et al. Meta-analysis of prognosis of different treatments for symptomatic moyamoya disease. World Neurosurg. 2019;127:354–61. doi: 10.1016/j.wneu.2019.04.062 30995556

[16] Yao Z, You C. Effect of surgery on the long-term functional outcome of moyamoya disease: a meta-analysis. Turk Neurosurg. 2019;29(2):171–9. doi: 10.5137/1019-5149.JTN.22598-18.3 29694660

[17] Kang S, Liu X, Zhang D, Wang R, Zhang Y, Zhang Q, et al. Natural course of moyamoya disease in patients with prior hemorrhagic stroke. Stroke. 2019;50(5):1060-6. 30909836 10.1161/STROKEAHA.118.022771

[18] Morioka M, Hamada J-I, Todaka T, Yano S, Kai Y, Ushio Y. High-risk age for rebleeding in patients with hemorrhagic moyamoya disease: long-term follow-up study. Neurosurgery. 2003;52(5):1049–54; discussion 1054-5. doi: 10.1227/01.neu.0000058223.73857.f4 12699546

[19] Sun H, Wilson C, Ozpinar A, Safavi-Abbasi S, Zhao Y, Nakaji P, et al. Perioperative complications and long-term outcomes after bypasses in adults with moyamoya disease: a systematic review and meta-analysis. World Neurosurg. 2016;92:179–88. doi: 10.1016/j.wneu.2016.04.083 27150649

[20] Zhang Q, Yin Z, Zhu C, Li W, Zhu H, Wang P, et al. Effectiveness of indirect revascularization for adult hemorrhagic moyamoya disease: a 10-year follow-up study. J Neurosurg. 2023:1–10. 10.3171/2023.6.JNS23727 37877987

[21] Cho W-S, Kim JE, Kim CH, Ban SP, Kang H-S, Son YJ, et al. Long-term outcomes after combined revascularization surgery in adult moyamoya disease. Stroke. 2014;45(10):3025–31. doi: 10.1161/STROKEAHA.114.005624 25184359

[22] Shamseer L, Moher D, Clarke M, Ghersi D, Liberati A, Petticrew M, et al. Preferred reporting items for systematic review and meta-analysis protocols (PRISMA-P) 2015: elaboration and explanation. BMJ. 2015;350:g7647. doi: 10.1136/bmj.g7647 25555855

[23] Kim C-Y, Wang K-C, Kim S-K, Chung Y-N, Kim H-S, Cho B-K. Encephaloduroarteriosynangiosis with bifrontal encephalogaleo(periosteal)synangiosis in the pediatric moyamoya disease: the surgical technique and its outcomes. Childs Nerv Syst. 2003;19(5–6):316–24. doi: 10.1007/s00381-003-0742-0 12743718

[24] Wang X, Liu H, Xu M, et al. Efficacy assessment of superficial temporal artery–middle cerebral artery bypass surgery in treating moyamoya disease from a hemodynamic perspective: a pilot study using computational modeling and perfusion imaging. Acta Neurochir. 2023;165(3):613-623. 36595057 10.1007/s00701-022-05455-9

[25] Zhu F-P, Zhang Y, Higurashi M, Xu B, Gu Y-X, Mao Y, et al. Haemodynamic analysis of vessel remodelling in STA-MCA bypass for moyamoya disease and its impact on bypass patency. J Biomech. 2014;47(8):1800–5. doi: 10.1016/j.jbiomech.2014.03.032 24720886

[26] Liu H, Song J, Xu M, Wang K, Ma L, Hu D, et al. Hemodynamic effects of tortuosity and stenosis in superficial temporal artery-middle cerebral artery bypass for moyamoya disease. World Neurosurg. 2024;186:e316–25. doi: 10.1016/j.wneu.2024.03.128 38548046

[27] Amin-Hanjani S, Singh A, Rifai H, Thulborn KR, Alaraj A, Aletich V, et al. Combined direct and indirect bypass for moyamoya: quantitative assessment of direct bypass flow over time. Neurosurgery. 2013;73(6):962–7; discussion 967-8. doi: 10.1227/NEU.0000000000000139 23949274

[28] Teo M, Abhinav K, Bell-Stephens TE, Madhugiri VS, Sussman ES, Azad TD, et al. Short- and long-term outcomes of moyamoya patients post-revascularization. J Neurosurg. 2022;138(5):1374–84. doi: 10.3171/2022.8.JNS22336 36272120

