# Supplementary material for: Long-term outcomes after revascularization surgery for adult moyamoya disease: Protocol for systematic review and meta-analysis
Source: PLoS One. 2025 Apr 17;20(4):e0318370. doi: 10.1371/journal.pone.0318370 (PMC12005512; doi:10.1371/journal.pone.0318370)
Supplement: S1 Appendix — (DOCX) [file pone.0318370.s001.docx]

**S1 Appendix. Detailed search strategies**

**Cochrane:**

(moyamoya disease) OR (moyamoya) OR (moya-moya) in Record Title AND (adult) OR (adults) in Title Abstract Keyword AND (outcome) OR (outcomes) OR (effectiveness) OR (efficacy) OR (rehabilitation) OR (prognosis*) OR (factor*) in Title Abstract Keyword AND "surgical" OR "surgery" OR "cerebral revascularization" OR "indirect bypass" OR "indirect revascularization" OR "direct bypass" OR "direct revascularization" OR "revascularization" OR "superficial temporal artery middle cerebral artery anastomosis" OR "encephalomyosynangiosis" OR "encephaloduroarteriosynangiosis" OR "encephaloduroarteriogaleosynangiosis" OR "encephaloduroarteriomyosynangiosis" OR "combined bypass" OR "direct anastomosis" OR "indirect anastomosis" OR "superficial temporal artery to middle cerebral artery" OR "STA-MCA" OR "STA-MCA bypass" OR "encephalo-duro-arterio-synangiosis" OR "EDAS" OR "encephalo-myo-synangiosis" OR "EMS" OR "encephalo-duro-arterio-myo-synangiosis" OR "multiple burr holes" OR "pial synangiosis" in All Text

**Ovid MEDLINE/EMBASE**

(moyamoya disease or moyamoya or moya-moya).ti. and (adult or adults).ab. and (outcome or outcomes or effectiveness or efficacy or rehabilitation or prognosis* or factor*).af. and ("surgical" or "surgery" or "cerebral revascularization" or "indirect bypass" or "indirect revascularization" or "direct bypass" or "direct revascularization" or "revascularization" or "superficial temporal artery middle cerebral artery anastomosis" or "encephalomyosynangiosis" or "encephaloduroarteriosynangiosis" or "encephaloduroarteriogaleosynangiosis" or "encephaloduroarteriomyosynangiosis" or "combined bypass" or "direct anastomosis" or "indirect anastomosis" or "superficial temporal artery to middle cerebral artery" or "STA-MCA" or "STA-MCA bypass" or "encephalo-duro-arterio-synangiosis" or "EDAS" or "encephalo-myo-synangiosis" or "EMS" or "encephalo-duro-arterio-myo-synangiosis" or "multiple burr holes" or "pial synangiosis").af.

**Web of Science**

(moyamoya disease) OR (moyamoya) or (moya-moya) (Title) AND adult* (Abstract) AND (outcome) OR (outcomes) OR (effectiveness) OR (efficacy) OR (rehabilitation) OR (prognosis*) OR (factor*) (All Fields) AND "surgical" OR "surgery" OR "cerebral revascularization" OR "indirect bypass" OR "indirect revascularization" OR "direct bypass" OR "direct revascularization" OR "revascularization" OR "superficial temporal artery middle cerebral artery anastomosis" OR "encephalomyosynangiosis" OR "encephaloduroateriosynangiosis" OR "encephatoduroarteriogaleosynangiosis" OR "encephaloduroarteriomyosinangiosis" OR "combined bypass" OR "direct anastomosis" OR "indirect anastomosis" OR "superficial temporal artery to middle cerebral artery" OR "STA-MCA" OR "STA-MCA bypass" OR "encephalo-duro-arterio-synangiosis" OR "EDAS" OR "encephalo-myo-synangiosis" OR "EMS" OR "encephalo-duro-arterio-myo-synangiosis" OR "multiple burr holes" OR "pial synangiosis" (All Fields)
